# Recent Studies of Artificial Intelligence on In Silico Drug Distribution Prediction

**DOI:** 10.3390/ijms24031815

**Published:** 2023-01-17

**Authors:** Thi Tuyet Van Tran, Hilal Tayara, Kil To Chong

**Affiliations:** 1 Department of Electronics and Information Engineering, Jeonbuk National University, Jeonju 54896, Republic of Korea; 2 Department of Information Technology, An Giang University, Long Xuyen 880000, Vietnam; 3 Vietnam National University–Ho Chi Minh City, Ho Chi Minh 700000, Vietnam; 4 School of International Engineering and Science, Jeonbuk National University, Jeonju 54896, Republic of Korea; 5 Advances Electronics and Information Research Center, Jeonbuk National University, Jeonju 54896, Republic of Korea

**Keywords:** ADMET, distribution prediction, drug discovery, artificial intelligence, machine learning, deep learning

## Abstract

Drug distribution is an important process in pharmacokinetics because it has the potential to influence both the amount of medicine reaching the active sites and the effectiveness as well as safety of the drug. The main causes of 90% of drug failures in clinical development are lack of efficacy and uncontrolled toxicity. In recent years, several advances and promising developments in drug distribution property prediction have been achieved, especially in silico, which helped to drastically reduce the time and expense of screening undesired drug candidates. In this study, we provide comprehensive knowledge of drug distribution background, influencing factors, and artificial intelligence-based distribution property prediction models from 2019 to the present. Additionally, we gathered and analyzed public databases and datasets commonly utilized by the scientific community for distribution prediction. The distribution property prediction performance of five large ADMET prediction tools is mentioned as a benchmark for future research. On this basis, we also offer future challenges in drug distribution prediction and research directions. We hope that this review will provide researchers with helpful insight into distribution prediction, thus facilitating the development of innovative approaches for drug discovery.

## 1. Introduction

**Figure 1:**
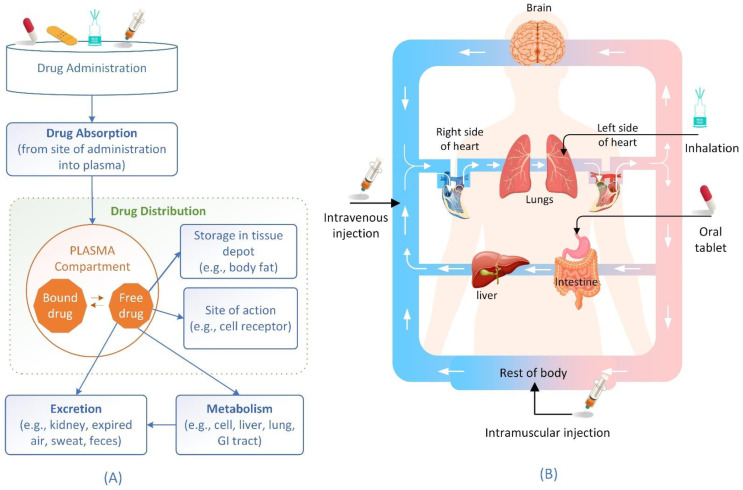
(**A**) Schematic description of the pharmacokinetic (ADME) process. (**B**) Drug administration and drug distribution process in the body [5].

Pharmacokinetics, the study of how pharmaceuticals are handled in the body, consists of four stages: absorption, distribution, metabolism, and excretion (ADME) (Figure 1A). It plays a very important role in drug research and development (R&D) because any drug candidate must be checked for pharmacokinetics and toxicity (ADMET) properties to ensure efficacy and safety. The average capitalized investment in R&D to bring a new medicine to the market is estimated at USD 1.1417 billion, after considering the cost of unsuccessful studies [1]. A key problem in drug R&D is the failure of compound candidates in clinical trials. Increasing success rates in clinical trials is believed to be the most profound factor in overall cost reductions and to outweigh savings in other phases [2]. If research improves the prediction of a drug’s failure by 10% before clinical trials, it could save about USD 100 million in development expenses for each drug [3]. Therefore, identifying chemical candidates with higher efficacy and no toxic or otherwise unfavorable side effects is a major challenge.

Early ADMET property assessment research may considerably increase the drug’s success rate, decrease the drug’s R&D costs, minimize the incidence of side effects and toxicities, and provide a direct therapeutic rationale for drug usage. Drug distribution is a critical step in the ADMET process because it has the potential to influence both the amount of drug that reaches the active sites as well as the effectiveness and toxicity of the drug. Lack of efficacy and uncontrolled toxicity are the main causes of 90% of medication failures during clinical development [4]. Drug distribution can cause unwanted reactions and side effects. Moreover, optimizing the distribution property affects other properties of ADMET because drug distribution is an important mediating process. Therefore, predicting drug distribution properties is essential during the early phases of drug research.

In traditional drug R&D, predicting distribution properties relies heavily on in vitro and in vivo studies. Despite progress in technological innovation, conventional experimental evaluations of distribution properties are often costly and time consuming. For example, assessing the blood–brain barrier penetration of every chemical takes one week and costs nearly USD 10,000 in a non-good laboratory practice facility [3]. Moreover, in vitro screening of compounds is typically limited to a few properties, with emphasis placed on only a few of the most promising chemical candidates. As a result, in silico distribution-related models are widely employed for quick and early screening of the drug distribution properties before they are further explored in vitro [6]. Because of the recent enormous success of artificial intelligence (AI) in many fields, AI-based drug R&D is ready to become a large force in the field of pharmaceuticals and is projected to make significant improvements in preclinical research. AI systems can efficiently and cheaply screen thousands or millions of candidate molecules rather than limiting the examination of distribution features to a select few. To construct, optimize, and improve the model performance, it is crucial to have a clear understanding of the distribution properties and the latest advances in AI-based distribution prediction models. Therefore, in this study, we comprehensively reviewed recent studies that used AI to predict drug distribution properties. Additionally, we collected available databases and datasets that the scientific community often uses for distribution prediction. We provided a list of free tools that support ADMET property prediction, along with the distributional property prediction performance of five recent tools. Finally, challenges and future directions for researchers working on AI-based distribution prediction are discussed. We believe that it will be helpful for researchers to work on improving the distribution property prediction model and other properties of the ADMET. It is important to note that the examination of drug distribution should be evaluated within the context of a particular drug delivery strategy. However, this manuscript primarily focuses on the application of AI-based models for predicting drug distribution.

## 2. Drug Distribution Process and Factors Affecting the Process

Once absorbed into the bloodstream, the medication circulates rapidly throughout the body. Drugs are transported from the bloodstream to body tissues during the recirculation process, which is the drug distribution stage. Drug distribution is the process by which an unmetabolized drug is distributed throughout the bloodstream and tissues of an organism (Figure 1B). The effectiveness or toxicity of medicine is dependent on its distribution in certain tissues, which explains in part why there is a lack of relationship between plasma levels and observed effects [7]. Drugs have varying distributions in various tissues, including fat, muscle, lungs, and brain, depending on their molecular structure and administration. The pharmacological effect of a drug depends on its concentration at the action site. This means that the distribution is a key factor in determining when, how strong, and sometimes how long the drug will work.

**Figure 2:**
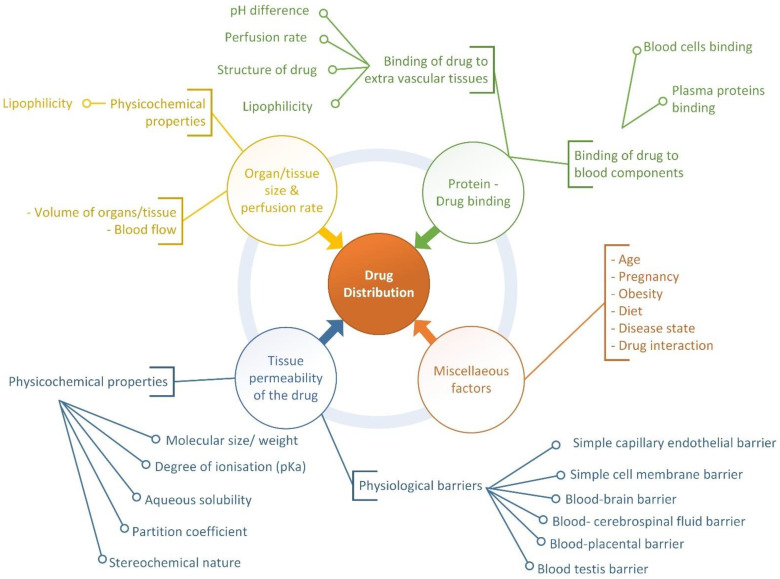
Factors influencing drug distribution.

There are two stages in the transport of medications from the bloodstream to the tissues outside the blood vessels: one, the rapid passage of free or unbound medication from the blood through the capillary wall and into the interstitial/extracellular fluid (ECF), and two, the passage of drug from the ECF across cell membranes to the intracellular fluid [8]. Various tissues absorb the drug from the plasma at various speeds and to varying degrees, leading to a non-uniform distribution of the drug throughout the body. Drug distribution is a passive process influenced by many factors, including drug permeability across tissues, organ/tissue size, perfusion rate, drug binding to tissue components, and other factors, as depicted in Figure 2. In preclinical in silico studies, the endpoints or properties of distribution were determined based on these factors. Based on large ADMET prediction systems and recent studies, we synthesized some properties (endpoints) of the distribution, such as physicochemical properties (molecular weight, heavy atoms, log P, log D, log S, pKa, etc.), plasma protein binding, blood–brain barrier, human placenta barrier, volume of distribution, fraction unbound in human plasma, and fraction unbound in the brain.

## 3. Performance Metrics to Evaluate and Compare AI-Based Distribution Prediction Methods

Evaluating the performance of AI methods is important to measure how effective a method is and to compare the performance of different methods fairly. In this review, we present the following evaluation metrics: accuracy (ACC), precision, recall, F1 score, area under the receiver operating characteristic curve (AUC), mean absolute error (MAE), root mean squared error (RMSE), coefficient of determination (R^2^), predictive relevance (Q^2^), and geometric mean fold error (GMFE). The formulas are as follows:

(1) $$ACC=\frac{TP+TN}{TP+TN+FP+FN}\;Range\left[0,1\right]$$

(2) $$Precision=\frac{TP}{TP+FP}\;Range\left[0,1\right]$$

(3) $$Recall=\frac{TP}{TP+FN}\;Range\left[0,1\right]$$

(4) $$F1score=\frac{2\times Precision\times Recall}{Precision+Recall}\;Range\left[0,1\right]$$

(5) $$\begin{array}{l} AUC=\\ Area\;under\;the\;receiver\;operating\;characteristic\;curve\;Range\left[0,1\right] \end{array}$$

(6) $$MAE=\frac{1}{N}\sum_{i=1}^{N}\left|y_{i}-\hat{y}\right|$$

(7) $$RMSE=\sqrt{\frac{1}{N}\sum_{i=1}^{N}{\left(y_{i}-\hat{y}\right)}^{2}}$$

(8) $$R^{2}=1-\frac{\sum {\left(y_{i}-\hat{y}\right)}^{2}}{\sum {\left(y_{i}-\overset{-}{y}\right)}^{2}}\;Range\left[0,1\right]$$

(9) $$Q^{2}=1-\frac{\sum {\left(y_{i}-\hat{y}\right)}^{2}}{\sum {\left(y_{i}-\overset{-}{y}\right)}^{2}}\;Range\left[0,1\right]$$

(10) $$GMFE=\frac{1}{N}\sum_{i=1}^{N}\left|\log_{10}\left(\frac{\hat{y}}{y_{i}}\right)\right|$$

In the Equations (1)–(3), TN, FN, TP, and FP represent the number of true negatives, false negatives, true positives, and false positives, respectively. In the Equations (6)–(10), *N*, $y_{i}$, $\hat{y}$, and $\overset{-}{y}$ represent total numbers of observation, the actual value for the ith observation, the predicted value of y, and mean value of y, respectively.

ACC is a measure of how many predictions made by a model are correct. It is calculated by dividing the number of correct predictions by the total number of predictions made. When the positive and negative classes are uneven, evaluating system performance based on accuracy may result in excessive bias [9]. In virtual screening data, the number of negative samples is frequently much greater than the number of positive ones. The accuracy score would exaggerate the performance of a failing prediction model that labels all cases as negative (i.e., inactive, or non-interacting). Precision is a measure of a model’s positive predictions’ accuracy. It is computed by dividing the number of accurate positive predictions by the total number of positive predictions generated by the model. Recall quantifies the proportion of actual positive cases that the model accurately identified. It is determined as the number of accurate positive predictions divided by the total number of actual positive cases. The F1 score quantifies the balance between recall and precision. It is computed based on the harmonic mean of precision and recall. AUC is a measure of the performance of a binary classification model. It is calculated by plotting the true positive rate against the false positive rate at various classification thresholds. The AUC ranges from 0 to 1, with higher values indicating better performance. MAE is a measure of the difference between predicted and actual values that is calculated by taking the mean of the absolute differences between the predicted and actual values. It is easy to understand and interpret, but it is not as sensitive to outliers as other measures. RMSE is calculated by taking the square root of the mean of the squared differences between the predicted and actual values. It is one of the most widely used measures of model performance, as it is easy to interpret and sensitive to both the mean and the variance of the error. R^2^ is a measure of how well a model fits the data. It is calculated as the ratio of the variance of the predicted values to the variance of the actual values. An R^2^ value of 1 indicates that the model perfectly fits the data, while a value of 0 indicates that the model does not fit the data at all. Q^2^ is a measure of the performance of a model in a predictive modeling context. It is calculated as the ratio of the variance of the predicted values to the total variance of the observed data. It ranges from −1 to 1, with a higher value indicating a better performing model. The main difference between R^2^ and Q^2^ is that R^2^ is a measure of model fit, while Q^2^ is a measure of model predictive ability [10]. R^2^ is calculated using the same data that was used to fit the model, while Q^2^ is calculated using a different set of data (i.e., a “holdout” set). This means that Q^2^ is a more conservative estimate of a model’s performance, as it reflects the model’s ability to generalize to new data. Another difference between R^2^ and Q^2^ is that R^2^ is only defined for regression models, while Q^2^ can be used for both regression and classification models. GMFE is calculated as the geometric mean of the fold errors, where the fold error is defined as the absolute value of the difference between the predicted and actual values. A lower GMFE indicates a better performing model.

It is worth noting that the results presented in the following sections are based on a specific dataset that may not have enough chemical diversity to accurately predict properties for new, first-in-class therapeutics.

## 4. AI-Based Distribution Property Prediction

AI is an area of computer science that aims to develop machines exhibiting human-like intelligence in problem solving, task performance, and learning [11]. Machine learning (ML) is a subset of AI involving the use of algorithms and statistical models to enable ML and improve its performance without explicit programming, which requires feeding the machine with large amounts of data and allowing it to learn patterns and relationships in the data. Deep learning (DL) is a specific type of ML that involves using neural networks designed to mimic the structure and function of the human brain. Deep learning algorithms are particularly effective on complex tasks and can process and analyze vast amounts of data and make decisions based on the patterns and relationships in the data. AI techniques can be used to analyze and predict potential ADMET properties of molecules, based on their structural and chemical properties. These techniques can be used to identify and prioritize molecules likely to exhibit good ADMET properties, which are important considerations during drug development. These techniques can also help reduce the time and cost of traditional testing methods by identifying molecules that are likely to have poor ADMET properties and removing them before clinical testing.

**Figure 3:**
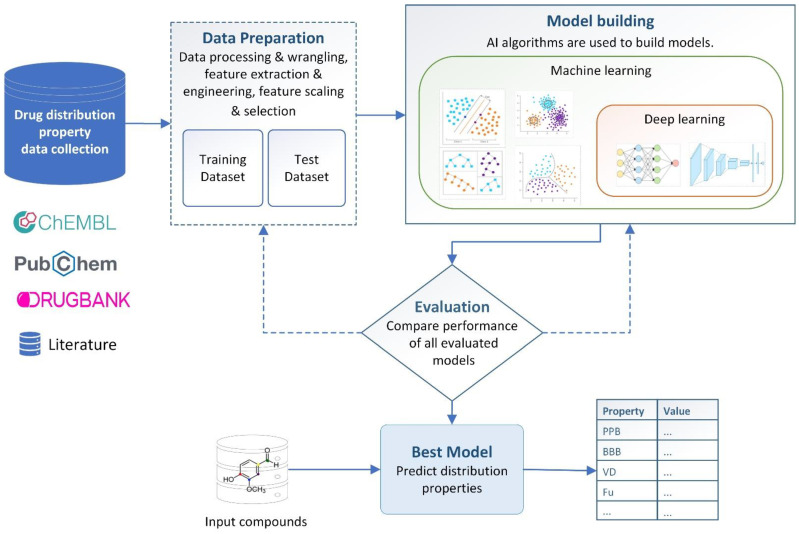
General structure of a drug distribution property prediction model using AI.

Drug distribution property prediction, notably AI-based drug distribution property prediction, has made great strides in recent years, allowing researchers to drastically reduce the time and money spent on eliminating unsuitable drug candidates. An overview of the general structure of an AI-based drug distribution property prediction model using the ML and DL approaches is presented in Figure 3. In this section, we focus on some properties such as plasma protein binding (PPB), fraction unbound in plasma (F_u_), blood–brain barrier (BBB), and volume of distribution (V_d_). For every distribution property, we discuss why it should be predicted and the evolution of AI-based studies that have predicted it in recent years.

### 4.1. Blood–Brain Barrier Permeability Prediction

BBB refers to the unique features of the central nervous system’s (CNS’s) microvasculature [12]. Drugs that target the CNS must penetrate the BBB to reach their molecular target. In contrast, drugs with peripheral targets may require minimal or no BBB penetration to avoid adverse CNS effects. Furthermore, the BBB plays an important role in protecting the brain parenchyma from blood-borne pathogens and significantly interferes with the entry of drug and other exogenous compounds into the CNS [13]. The logarithmic ratio of drug concentrations in the brain and blood, log BB, is the most commonly used quantitative measure of a molecule’s capability [14]. Experiments in vivo to determine log BB are complex and time consuming; the log BB data obtained are usually small with no high reliability. To overcome these disadvantages, recent AI-based BBB permeability prediction methods have focused on classifying whether a given compound is BBB permeable (BBB+) or not (BBB-) rather than on the log BB data.

Recently, AI-based approaches have increasingly been used to predict of BBB permeability (Table 1). Some commonly used ML algorithms, such as random forest (RF), support vector machine (SVM), k-nearest neighbor (k-NN), decision trees (DT), gradient boosting (GB), and extreme boosting system (XGB), achieve a prediction accuracy higher than 80% [15,16,17,18,19]. Notably, the LightBBB server [16] was developed by Shaker et al. They used the light gradient boost machine (LightGBM) algorithm, a large dataset of 7162 compounds collected from previous studies and 2432 1D and 2D descriptors. Using 10-fold cross-validation, they achieved an ACC of 89% and an AUC of 93%. This performance was lower than that of the mixed DL model on the same dataset, with an ACC of 92% and an AUC of 96% [20]. However, they built a useful BBB prediction tool, that is available at http://ssbio.cau.ac.kr/software/bbb (accessed on 15 October 2022). Two ML-based ADMET predictors, admetSAR 2.0 [21] and FP-ADMET [22], also achieved very high BBB prediction results with an ACC of 90.7% and 81%, respectively.

In the last few years, several researchers have presented various DL algorithms for BBB permeability prediction with excellent results, such as artificial neural networks (ANN), deep neural networks (DNN), convolutional neural networks (CNN), recurrent neural networks (RNN), and graph convolutional neural networks (GCNN) [23,24,25,26,27,28,29,30,31]. Alsenan et al. used an RNN model to improve the accuracy of BBB permeability prediction [29]. The dataset for their experiment contains 2350 compounds collected from Wang et al. [32] and 6394 descriptors and fingerprints for each compound. With an ACC of 96.53% and an AUC of 98.6%, the obtained results demonstrated that their RNN model solved the three identified issues of the previous BBB prediction model: imbalanced datasets, high dimensionality, and enhanced classifier performance. Their DL model also achieved the same performance using the same dataset [30].

Despite their outstanding performance, these models have a problem in common with other AI-based models: lack of interpretability [27]. This nature of the “black box” does not help researchers learn how to better design CNS drugs. To overcome this drawback, Yu’s research team developed a method to combine the strengths of ML and DL to produce a set of simple rules that are simple to understand and make predictions with better accuracy [27]. This is a hybrid method between the SVM and GCNN algorithms using a dataset of 940 drugs on the market and eight optimum descriptors with the highest essential scores. As a result, the novel hybrid ensemble model performed better than other traditional constitutive quantitative structure–activity relationship (QSAR) models, with an ACC of 96% and an AUC of 98%. This hybrid model is not limited to CNS drug prediction but can also be used for other ADMET property predictions.

AI-based BBB permeability prediction models use smaller datasets and fewer features, which are often less reliable because they do not cover sufficient chemical diversity [16]. Therefore, reliable datasets that are sufficiently large to build models should be chosen.

**Table 1:** Summary of recent AI-based studies predicting BBB property.

Method	Data Sources	No. of Compounds	Performance	Ref.
SVM, RF, XGB	[32,33,34,35]	1970	AUC = 0.957, ACC = 0.910	[15]
LightGBM	[17,18,32,36,37,38,39,40]	7162	AUC = 0.94, ACC = 0.89	[16]
Mixed DL: Multilayer Perceptron (MLP), CNN	[16]	7162	AUC = 0.96, ACC = 0.92	[20]
RF, MLP, Sequential Minimal Optimization	[33,36,41]	2313	ACC = 0.88	[17]
Logistic Regression, DT, RF, GB	[42]	968	AUC = 0.78, ACC = 0.817	[18]
SVM, k-NN, DT, DNN	SIDER [40,43]	1000	ACC = 0.97, AUC = 0.98	[19]
Multichannel Substructure-Graph Gated Recurrent Unit Architecture	[37]	2053	AUC = 0.753	[23]
CNN	[37]	2039	AUC = 0.694	[24]
CNN	[35,44]	2254	ACC = 0.755, AUC = 0.784	[25]
CNN	[15,16,18,36,37,40]	7224	ACC = 0.74, AUC = 0.83	[45]
ANN	[46,47]	300	RMSE = 0.171	[26]
SVM and GCNN	[48]	940	ACC = 0.96, F1 score = 0.95	[27]
Fully Connected Neural Network, CNN	[37,40]	2264	AUC = 0.995	[28]
RNN	[32]	2350	ACC = 0.965, AUC = 0.98	[29]
DNN	[32]	2350	ACC = 0.962, AUC = 0.968	[30]
XGraphBoost	[38,49]	2039	AUC = 0.932	[31]

### 4.2. Plasma Protein Binding Prediction

**Figure 4:**
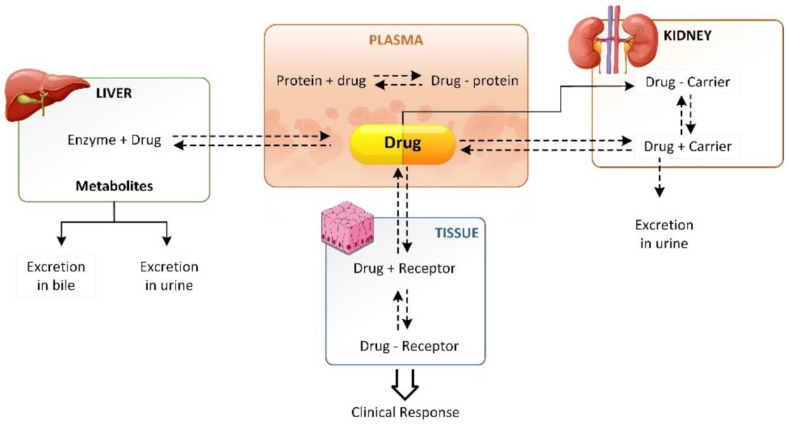
Relationship of reversible drug–protein binding in the plasma, drug distribution, and elimination.

One of the main mechanisms of drug absorption and distribution is through PPB. Therefore, drug binding to plasma proteins has a strong effect on the pharmacodynamic activity of drugs. PPB can directly affect oral bioavailability because free drug concentrations are at stake when the drug binds to serum proteins [50]. Figure 4 depicts the bi-dimensional interaction between drug–protein binding in the plasma, drug distribution, and drug elimination [51]. Many in silico predictive PPB models using ML and DL have been constructed using various datasets and evaluation units (Table 2). In this sense, in silico approaches can be cost effective, quick, and potent for screening large amounts of molecules, even without the need to synthesize the substance, as its structure suffices [52].

In an in silico strategy, Yuan et al. developed a QSAR model for predicting human PPB on a large dataset that was collected and curated from multiple studies over the past 15 years with 6741 compounds [53]. The QSAR model for different levels was constructed for the three corresponding descriptor sets: ADMET, Dragon, and the PaDEL, using five ML algorithms: RF, support vector regression (SVR), k-NN, boost tree (BT), and gradient-enhanced regression (GER). The best performance of their model is much higher than that of the previous models, with an MAE of 0.076 on the test set and an MAE falling to 0.041 at high binding (PPB > 0.8), 0.127 at moderate binding (PPB = 0.4–0.8), and 0.156 at low binding (PPB < 0.4). Their models performed well in the external evaluation set, which included 99 compounds from traditional Chinese medicine with an MAE value of 0.149.

Recently, Venkatraman et al. used the RF algorithm based on the fingerprint to predict PPB for 8103 compounds, achieving a balanced ACC of 84% and an AUC of 92% [22]. Xiong and co-authors developed an ADMET prediction-free web tool using the multitask graph attention framework [50]. They used 4712 compounds to predict PPB, and their model yielded a high-accuracy prediction with an R^2^ of 0.733 and an RMSE of 0.135. An enhanced Graph Isomorphism Network (MolGIN) was proposed by Peng et al., utilizing the bond features and distinctions in the impact of atom neighbors for predicting ADMET properties [54]. The PPB dataset was collected by Wang et al. [55] contained 1830 compounds. The test results for an R^2^ of 0.738 show that MolGIN is significantly superior to other baseline models (RF, graph neural network (GNN), DNN) in terms of efficiency measurement and achieves performance comparable to or superior to modern models (admetSAR 2.0 [21], ADMETLab 1.0 [6]) on the same dataset.

In 2022, Lou et al. proposed a new strategy for predicting and optimizing the human BBB for substances using an interpretable DL approach [56]. They used the attentive fingerprint algorithm, 3921 compounds, and Morgan fingerprints to develop an interpretable DL model. With an RMSE of 0.112 on the test set, their model showed promising predictive ability. Moreover, it could offer lead compounds with particular structural change plans to improve the PPB properties, unlike conventional QSAR models. Interpretable DL approaches allow us to understand why the model generates such predictions, helping us comprehend chemical pathways and rationally construct a structural change scheme.

Other PPB prediction studies are summarized in Table 2. In general, most models are built on different datasets; therefore, their performances cannot be compared with each other. Nevertheless, they will be helpful tools for assessing PPB during the process of drug design or structural modification.

**Table 2:** Summary of recent AI-based studies predicting PPB property.

Method	Data Sources	No. of Compounds	Performance	Ref.
RF	[57]	670	R^2^ = 0.74, RMSE = 0.12	[52]
RF	[53,58]	8103	ACC = 0.84, AUC = 0.92	[22]
SVM	AstraZeneca in-house	100,550	RMSE = 0.444, R^2^ = 0.721	[59]
k-NN, SVR, RF, BT, and GER	[55,60,61,62,63,64,65,66,67,68,69], CHEMBL and DrugBank	6741	MAE = 0.076	[53]
GCNN	[62]	1209	R^2^= 0.668, RMSE = 0.191	[21]
Multitask graph attention framework	ChEMBL, PubChem, OCHEM, Literature	4712	R^2^ = 0.733, RMSE = 0.135	[50]
GNN	[61,62]	1744	R^2^ = 0.747	[70]
MolGIN method	[55]	1830	R^2^ = 0.738	[54]
GCNN, GAT	ChEMBL, PubChem, DrugBank, Literature	1830	R^2^ = 0.563, RMSE = 0.211	[71]
Attentive fingerprint algorithm (GNN)	[56]	3921	R^2^ = 0.841, RMSE = 0.112	[56]

### 4.3. Fraction Unbound in Plasma Prediction

In pharmacodynamic and pharmacokinetic studies, the F_u_ is a critical determinant of therapeutic efficacy. Most drugs in plasma are in an equilibrium state between unbound and bound to serum proteins [50]. The unbound fraction of the drug diffuses into tissues and is metabolized, or eliminated from the body [72]. In other words, only this fraction can be transferred to the sites of action across the membranes, whereas the bound fraction acts as a reservoir for the free drug concentration and prolongs the duration of action [73]. Distinct pharmacokinetic effects were observed as this fraction varied. The degree to which a drug attaches to proteins in the bloodstream may impair its efficacy; the more bound it is, the less efficiently it may pass cellular membranes or diffuse. F_u_ influences the renal glomerular filtration rate and hepatic metabolism. As a result, it affects the drug’s volume of distribution and total clearance, both of which are critical elements in determining its pharmacokinetics [74]. Consequently, it is critical to make an accurate estimate of the F_u_ of drug candidates, particularly in low-value regions, throughout the drug development process. This section summarizes the progress of AI-based F_u_ prediction studies since 2019 (see Table 3).

Venkatraman investigated the efficiency of fingerprint-based RF models for predicting many ADMET-related properties [22]. In particular, his F_u_ prediction method achieved comparable or better performance compared with traditional 2D and 3D molecular descriptors on 2391 compounds with an R^2^ of 0.63 and an RMSE of 0.44. The QSAR model was constructed by Wang et al. using several highly efficient, powerful, and widely used ML methods such as RF, SVM, GB, and XGB [75]. They used a dataset of 1352 drugs from a previous study [76], and the results once again proved that the RF model has a superior predictive power to the other methods in predicting F_u_, with an R^2^ of 0.818 and an RMSE of 0.291. Recently, Mulpuru and Mishra used a freely available automated ML framework, including AutoKeras, PyCaret, Auto-Sklearn, and TPOT, with chemical fingerprints to build F_u_ predictions [77]. Their best prediction model on a large dataset of 5471 compounds from ChEMBL was impressive, with an R^2^ of 0.85, giving their model a significant contribution to ADMET modeling.

In addition to the ML techniques, DL techniques have also been exploited by many authors with the goal of improving prediction efficiency on large datasets. Zhou et al. built a QSAR model using DNNs and chemical fingerprints on 24 industrial ADME datasets from Lilly’s in-house ADME assay with 9730 molecules to train the F_u_ prediction model [78]. However, the comparison results showed that their DNN model was not better than the SVM model on the same dataset, with an RMSE of 0.086 (SVM: RMSE = 0.083). In another study, Feinberg et al. built an ADMET prediction model with multitask deep featurization using a GCNN on a large dataset of 13,388 training compounds and 4462 testing compounds [79]. Their prediction model achieved significantly higher prediction accuracy than with the RF model, which had an R^2^ of 0.919 (RF: R^2^ = 0.582).

**Table 3:** Summary of recent AI-based studies predicting F_u_ property.

Method	Data Sources	No. of Compounds	Performance	Ref.
SVM, RF, GB, XGB	[76]	1352	R^2^ = 0.82, RMSE = 0.291	[75]
AutoML Framework	ChEMBL v.27	5471	R^2^ = 0.85, RMSE = 8.44	[77]
QSAR/Partial Least Squares (PLS) model	[69,80]	599	Q^2^ = 0.69	[81]
DNNs	ADMET assays	9730	RMSE = 0.086	[78]
PotentialNet GCNNs	ADMET assays	17,850	R^2^ = 0.919	[79]

### 4.4. Volume of Distribution Prediction

**Figure 5:**
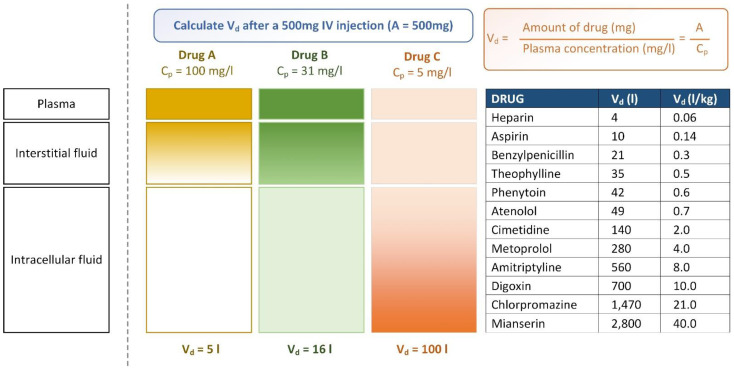
Examples of volume of distribution. Adapted from Maxwell [85].

The volume of distribution (V_d_) is a pharmacokinetic measure that indicates how long a drug will remain in the plasma or whether it will redistribute to other tissue compartments [82]. In other words, V_d_ is a theoretical concept of the dose used with actual initial concentrations in circulation, and it is a critical property for describing drug distribution in the human body [50]. V_d_ affects the half-life and duration of the activity of the compound at a steady state [83]. When two drugs have the same daily dose, the one with a lower V_d_ (shorter half-life) may require more frequent dosing (at lower individual doses) to attain a pharmacodynamic profile comparable to that with a higher V_d_ at a steady state. V_d_ is also a critical pharmacokinetic metric for determining the plasma concentration–time profile and half-life of drugs [84]. Figure 5 illustrates how to calculate V_d_ when we use three different drugs (A, B, and C) at a dose of 500 mg in an intuitive manner [85]. In addition, the table in Figure 5 shows the V_d_ values of some commonly used drugs. Several AI-based models have been successful in predicting V_d_ (Table 4).

Based on the ML technology, three authors used RF algorithms to develop V_d_ prediction models and made significant contributions in the past year [22,59,86]. Especially, the FP-ADMET prediction software was developed by Venkatraman [22]. This is a powerful tool for ADMET prediction based on fingerprints. The efficiency of the V_d_ prediction model for 1951 compounds were R^2^ = 0.45 and RMSE = 0.51. AstraZeneca has nearly 20 years of development experience in AI-based ADME models [59]. AstraZeneca’s in-house data and models are updated regularly, and their accuracy increased over time. Their V_d_ prediction model achieved high accuracy with an R^2^ of 0.67 and an RMSE of 0.371. Simeon et al. constructed a QSAR model for predicting V_d_ in humans, rats, dogs, mice, and monkeys using RF, PLS, and ANN algorithms [87]. Their models were built using physicochemical descriptors, electronic state descriptors, fingerprint descriptors, or a combination of physicochemical descriptors and one of the other two descriptors. Using the V_d_ human dataset of 1442 compounds, the RF model had a highly accurate prediction on the test set with an R^2^ of 0.61 and an RMSE of 0.41.

In another study, Wang et al. used four ML algorithms, RF, SVM, GBM, and XGB, to develop a quantitative property–structure relationship (QSPR) model to predict V_d_ [75]. Their models were assessed by 10-fold cross-validation on a dataset containing 1352 drugs from Lomabardo et al. [76] using 209 selected features. The best-performing model was the SVM model, with an R^2^ of 0.870 and an RMSE of 0.208. A new model called DeepPharm, using integrated transfer learning and multitask learning approaches, was developed by Ye et al. [88]. DeepPharm is more efficient than conventional ML methods such as PLS regression, SVM, ANN, RF, and k-NN on 412 molecules from the FDA, with an accuracy of 63.33% and a MAE of 0.175. To improve ADMET prediction, Feinberg and co-authors proposed a multitask deep featurization method applying GCNN using a large dataset containing 45,229 compounds for training and 15,076 compounds for testing [79]. However, compared with the RF method on the same dataset, this method did not improve significantly, with an R^2^ of 0.525 (RF: R^2^ = 0.520).

**Table 4:** Summary of recent AI-based studies predicting V_d_ property.

Method	Data Sources	No. of Compounds	Performance	Ref.
RF	[89]	1303	GMFE = 2.15 % < 2-fold = 54 % < 3-fold = 73	[86]
RF	AstraZeneca in-house	1440	RMSE = 0.371, R^2^ = 0.67	[59]
SVM, RF, GB machine, XGB	[76]	1352	R^2^ = 0.87, RMSE = 0.208	[75]
PLSANN, RF	ChEMBL [76,90]	1442	R^2^ = 0.61, RMSE = 0.41	[87]
PLS regression, SVM, ANN, RF, k-NN, multitask learning feed-forward neural network, DeepPharm	Drugbank	412	ACC = 0.63, MAE = 0.174	[88]
PotentialNet GCNNs	ADMET assays	63,305	R^2^ = 0.525	[79]

## 5. Public AI-Based ADMET Prediction Tools

With the continual collection of experimental ADMET data in recent years, many AI-based prediction tools for diverse endpoints have been developed to efficiently facilitate ADMET evaluation. More specifically, they can help researchers evaluate the ADMET properties in a time and money-saving manner, screen for undesirable compounds, and gather timely feedback on ADMET information for lead optimization. Moreover, they are also good support for distribution prediction researchers in developing and improving models. In Table 5, we list popular AI-based ADMET prediction tools that have been newly developed or updated in the last few years. These tools have built-in drug distribution property predictions and are available for free.

In particular, we are interested in five publicly available ADMET predictors developed from 2019 to 2022, namely AdmetSAR 2.0 [21], ADMETLab 2.0 [50], FP-ADMET [22], Interpretable-ADMET [71], and HelixADMET [70]. They predicted most of the main ADMET-related properties (from 50 to 67 endpoints) and demonstrated good predictive performance. In Table 6, we analyze the predictive performance across the four distribution properties. These free and user-friendly tools can help ADMET researchers quickly and easily identify ADMET profiles for a wide range of drug candidates. Furthermore, these tools can serve as benchmarks for future ADMET studies. More interestingly, the Interpretable-ADMET tool helps optimize drug candidates with undesirable ADMET properties by automatically creating a new set of virtual candidates based on matching molecular pair rules.

Overall, practical applications demonstrate that the tool is limited to qualitative analysis of chemicals and cannot accurately anticipate the quantitative values of certain properties [95]. Moreover, most reports indicate that these tools achieve very high or acceptable prediction accuracies. However, most predictive data have considerable uncertainty, and the decision is sensitive to a particular property [96]. We should choose tools with a larger amount of training data, higher accuracy, and higher citations, and use various tools to analyze data to make more accurate decisions.

## 6. Data Sources for Distribution Prediction Research

The success of an AI-based predictive model is highly dependent on the data and the modeling approach. The availability of an increasing number of public datasets on human pharmacokinetics facilitates the collection of a large number of structural substances and their associated experimental values for modeling purposes. A thorough understanding of the origin and reliability of the data is essential. Table 7 summarizes the most popular data sources for ADMET prediction research, specifically AI-based distribution prediction. In addition, researchers in the field of distribution prediction often use multiple datasets aggregated from different studies to develop and test their models. Interested readers may refer to the additional data sources from the literature provided in Section 3. Although the data sources for the study predicting the distribution were numerous, their quality was insufficient. Therefore, when using any source of test data to build a model, experts must carefully evaluate the certainty and reliability of the test. We should select reliable data sources, aggregate them, and use datasets that are sufficiently large for model training. Sharing more experimental data from pharmaceutical companies would be helpful to the scientific community. We hope that further development of big data will bring promising prospects for future drug distribution research.

## 7. Challenges for AI-Based Distribution Prediction Researcher

The increasingly powerful AI technology in drug R&D presents not only many opportunities but also many challenges for researchers in predicting drug distribution and ADMET properties.

The first challenge is the lack of data quality [95,108,109,110,111,112,113]. Public data sources for drug R&D are undeniably increasing significantly; however, AI algorithms need not only the quantity of data but also the quality of data that are high enough to make accurate models. The chemicals tested should be sufficiently diverse to allow generation methods to cover the entire chemical search space [114]. Therefore, to solve this problem, it is important to collect high-quality data. Experts argue that more empirical data are required to create higher quality models and maximize the potential of AI-based applications [109]. However, in vivo and in vitro data collection is complex and limited [115]. Other problems related to the variability of the experiment, such as errors occurring in the process of data collection, management, and manipulation, also affect the quality of data. The statistical challenges and combination of diverse data with varying noise and bias are significant. Therefore, extracting and collecting high-quality data to train computational models is a laborious and challenging task that must be performed by experts. Recently, data sources from fields such as biology, chemistry, pharmacology, and clinical trials have been collected to build “big data” for drug R&D [116]; however, many obstacles still exist. Technical challenges, such as missing data, dimensional inaccuracies, and bias control, make big data analytics complex [117]. More time is needed to build a complete big data system for drug discovery and development. Meanwhile, there are many useful sources of data from proprietary pharmaceutical companies that are yet to be shared publicly with the research community [118]. Therefore, security and reasonable sharing policies are essential and contribute to solving data difficulties during this period.

The second challenge is model quality. In addition to data quality, a suitable learning model is required to harness the power of AI to predict distributional properties. First, it requires researchers to have extensive knowledge of building AI-based models such as ML and DL algorithms. Without the expertise needed to build an effective data-mining project, researchers sometimes rely on incorrect methods that can lead to common errors or overly optimistic results [119]. AI-based models are evolving rapidly, and their complexity is increasing exponentially, requiring researchers to grasp new techniques quickly. Additionally, the current data landscape also necessitates the creation of powerful novel computational approaches capable of accurately predicting outcomes with diverse, large, multidimensional, and sparse data.

The third challenge faced by researchers is the difficulty in understanding the nature of AI models. Although the performance of AI-based distributed property prediction models is impressive, the mechanistic interpretation is still lacking. Therefore, it is difficult for scientists to assess the novelty or reliability of the hypothesis generated by AI because of its black-box nature, which hinders the improvement of the model and the optimization of compounds with undesirable distribution properties.

As in drug discovery, drug distribution is multidisciplinary. To study and build predictive models of distribution characteristics, researchers need to equip themselves with relevant knowledge in areas such as biology, bioinformatics, pharmacology, chemistry, and chemical informatics [120]. This is a big challenge for independent researchers. In fact, programmers and modelers who analyze huge datasets and build AI models often have a theoretical background and are ignorant of data-generating experiments and their flaws. AI experts are rarely chemists or biologists, especially structural representation specialists. Therefore, identifying potential mistakes in large datasets and interpreting the results of AI models remains difficult. Researchers must understand drug properties, endpoint roles, effects, metrics and assessments, structure–exposure–activity relationships, drug interactions, and other related knowledge aspects. Not all distribution properties are detrimental to all medicines. For instance, medications that target disorders of the central nervous system must typically be able to cross the BBB, although this trait is generally absent in other diseases. Therefore, collaboration between scientists is essential to ensure the correctness, effectiveness, and usability of drug property prediction models.

## 8. Conclusions and Future Perspectives

The application of AI to improve the drug R&D process is still in its infancy at this point [121]. For AI to reach the pinnacle of drug R&D, time and effort are required from multidisciplinary researchers. This is both an opportunity and a challenge for researchers. An accurate prediction of the distribution property is an important part of determining the ADMET profile of a drug candidate. Optimization of the distribution properties has a direct influence on the effectiveness and toxicity of the drug. Despite data challenges, the current efforts of AI-based, distributed property prediction models have made positive contributions, such as reducing costs and time in drug R&D.

One approach to overcome the challenges in AI-based drug distribution prediction is to focus on improving the quality and quantity of the data used for training and testing the models. This can be achieved by incorporating a wider range of data sources, including clinical trials, electronic health records, and real-world evidence. Additionally, implementing advanced data-cleaning and preprocessing techniques can help reduce the noise and bias in the data, leading to more accurate and reliable predictions. Another direction for future research in this field is the development of more sophisticated and robust AI algorithms that can handle complex and dynamic data. This includes the use of DL techniques, which have shown promising results in various medical and health applications. Additionally, it is crucial to ensure the interpretability of AI-based drug distribution predictions, which can be achieved by developing methods to visualize and interpret the underlying mechanisms and processes behind the model’s predictions, thus, increasing trust and confidence in the results and enabling good decision making in drug development and personalized medicine. Furthermore, it is essential to incorporate human expertise and domain knowledge into the development and evaluation of AI-based drug distribution prediction models. This can be achieved through collaboration between AI researchers and medical experts, such as pharmacologists and clinicians, to ensure that the models align with existing knowledge and practices in the field.

The core problem of AI systems is the process of “learning”. Good learning requires high-quality data and a high-quality learning model. “Quality” requires expert supervision. In the future, drug R&D data for learning will increase rapidly in quantity and complexity, requiring powerful AI-based predictive models such as DL. Ensuring the correctness and effectiveness of an AI-based predictive model requires close collaboration between multidisciplinary scientists. The contribution and sharing of data from pharmaceutical companies and academic researchers will accelerate the development of big data. In the next decade, data, computation, and multidisciplinary scientists will become highly connected to AI-based drug R&D. There is a continuous feedback loop between interpretable AI and experimental biology. Through incremental improvements to workflow and comprehensible insights, researchers can track, evaluate, and construct better prediction models.

In this study, the recent development of AI-based distribution property prediction models were analyzed and synthesized. Although each model has its own limitations, the models show a remarkable effort by researchers. The basics of distribution and the role of endpoints, along with related resources such as public data sources and free prediction tools, are provided. We hope that this is a useful document for researchers who develop and improve distribution property prediction models and other properties of ADMET based on AI.

## Figures and Tables

**Table 5:** Public AI-based ADMET prediction tools.

Name	No. of ADMET Prediction Models	Methods	Website *	Ref.
OECD QSAR Toolbox	902	QSAR	https://qsartoolbox.org/	[91]
iDrug ADMET prediction	60	AI	https://drug.ai.tencent.com/console/en/admet	
AdmetSAR 2.0	52	RF, SVM, k-NN	http://lmmd.ecust.edu.cn/admetsar2/	[21]
ADMETlab 2.0	67	GNN	https://admetmesh.scbdd.com/	[50]
Interpretable-ADMET	59	GCNN GAT	http://cadd.pharmacy.nankai.edu.cn/interpretableadmet/	[71]
HelixADMET	52	RF, GNN	https://paddlehelix.baidu.com/app/drug/admet/train	[70]
FP-ADMET	50	RF	https://gitlab.com/vishsoft/fpadmet	[22]
SwissADME	35	MLR, SVM, RNN, etc.	http://www.swissadme.ch/	[92]
vNN-ADMET	15	k-NN	https://vnnadmet.bhsai.org/	[93]
ICDrug ADMET	14	RF	www.icdrug.com/ICDrug/ADMET	[94]
Virtual Rat	12	RF, C5.0, DT	https://virtualrat.cmdm.tw/	[3]
LightBBB	1 (BBB)	Light GBM	http://ssbio.cau.ac.kr/software/bbb	[16]
Deep B^3^	1 (BBB)	CNN	http://cbcb.cdutcm.edu.cn/deepb3/	[45]

* The websites were accessed on 10 October 2022.

**Table 6:** Performance of five ADMET prediction tools on distribution property prediction.

Property	Tool	Methods	No. of Compounds	Performance	
				AUC	R^2^
BBB	AdmetSAR 2.0	SVM	1839	0.944	
	ADMETLab 2.0	GNN	1601	0.908	
	FP-ADMET	RF	7236	0.92	
	Interpretable-ADMET	GCNN & GAT	1830	0.897	
	HelixADMET	GNN	1791	0.944	
PPB	AdmetSAR 2.0	GCNN	1209		0.668
	ADMETLab 2.0	GNN	1573		0.733
	FP-ADMET	RF	8103	0.92	
	Interpretable-ADMET	GCNN & GAT	2044		0.563
	HelixADMET	GNN	1744		0.747
F_u_	AdmetSAR 2.0	-	-	-	-
	ADMETLab 2.0	GNN	1494		0.763
	FP-ADMET	RF	2319		0.63
	Interpretable-ADMET	-	-	-	-
	HelixADMET	-	-	-	-
V_d_	AdmetSAR 2.0	-	-	-	-
	ADMETLab 2.0	GNN	1399		0.782
	FP-ADMET	RF	1951		0.45
	Interpretable-ADMET	-	-	-	-
	HelixADMET	-	-	-	-

**Table 7:** Public data sources for AI-based distribution prediction research.

Name	Data Size (Compounds) *	Website *	Ref.
ZINC20	>750 million	https://zinc20.docking.org/	[97]
ChemSpider	115 million	http://www.chemspider.com/	[98]
PubChem	>111 million	https://pubchem.ncbi.nlm.nih.gov/	[99]
Therapeutics Data Commons	4,264,939	https://tdcommons.ai/	[100]
OCHEM 4.2	3,791,680	https://ochem.eu/home/show.do	[58]
openFDA	>3 million	https://open.fda.gov/	[101]
ChEMBL	>2.2 million	www.ebi.ac.uk/chembl/	[102]
GOSTAR	1.76 million	https://www.gostardb.com/	
BindingDB	>1 million	https://www.bindingdb.org/	[103]
Supernatural II	325,508	http://bioinformatics.charite.de/supernatural	[104]
NIST Chemistry WebBook	>70,000	http://webbook.nist.gov/	[105]
SIDER 4.1	55,730	http://sideeffects.embl.de/	[43]
ContaminantDB	>54,000	https://contaminantdb.ca/	
DrugBank 5.1.9	14,665	http://www.drugbank.ca/	[106]
IMPPAT 2.0	17,967	https://cb.imsc.res.in/imppat	[107]
KEGG	12,000	https://www.kegg.jp/	

* Data size and websites were accessed on 10 October 2022.

## Data Availability

Not applicable.
